# TIM3 Mediates T Cell Exhaustion during *Mycobacterium tuberculosis* Infection

**DOI:** 10.1371/journal.ppat.1005490

**Published:** 2016-03-11

**Authors:** Pushpa Jayaraman, Miye K. Jacques, Chen Zhu, Katherine M. Steblenko, Britni L. Stowell, Asaf Madi, Ana C. Anderson, Vijay K. Kuchroo, Samuel M. Behar

**Affiliations:** 1 Department of Microbiology and Physiological Systems, University of Massachusetts Medical School, Worcester, Massachusetts, United States of America; 2 Department of Neurology, Brigham and Women's Hospital and Harvard Medical School, Boston, Massachusetts, United States of America; Portland VA Medical Center, Oregon Health and Science University, UNITED STATES

## Abstract

While T cell immunity initially limits *Mycobacterium tuberculosis* infection, why T cell immunity fails to sterilize the infection and allows recrudescence is not clear. One hypothesis is that T cell exhaustion impairs immunity and is detrimental to the outcome of *M*. *tuberculosis* infection. Here we provide functional evidence for the development T cell exhaustion during chronic TB. Second, we evaluate the role of the inhibitory receptor T cell immunoglobulin and mucin domain–containing-3 (TIM3) during chronic *M*. *tuberculosis* infection. We find that TIM3 expressing T cells accumulate during chronic infection, co-express other inhibitory receptors including PD1, produce less IL-2 and TNF but more IL-10, and are functionally exhausted. Finally, we show that TIM3 blockade restores T cell function and improves bacterial control, particularly in chronically infected susceptible mice. These data show that T cell immunity is suboptimal during chronic *M*. *tuberculosis* infection due to T cell exhaustion. Moreover, in chronically infected mice, treatment with anti-TIM3 mAb is an effective therapeutic strategy against tuberculosis.

## Author Summary

Tuberculosis is a leading cause of morbidity and mortality across the globe. Fortunately, most people infected with *M*. *tuberculosis* mount a protective immune response and only a small fraction develops active disease. Impairment of immunity late during the course of disease can lead to bacterial recrudescence; however, why immunity fails is poorly understood. We investigated whether T cell exhaustion develops and contributes to immunological impairment during disease. Our studies provide definitive evidence that CD4^+^ T cells become functionally exhausted early after infection, and subsequently, CD8^+^ T cells also show signs of dysfunction. T cell exhaustion in both subsets was associated with the expression of multiple inhibitory receptors. Exhausted T cells expressed TIM3 plus other inhibitory receptors (e.g., PD1, TIM3, Lag-3, and 2B4), TIM3^+^PD1^+^ T cells were more likely to be poor producers of IL-2, IFNγ, and TNF and instead produce IL-10. Evaluation of gene expression by Nanostring confirmed that TIM3^+^PD1^+^ T cells in the lungs of infected mice had a transcriptional profile characteristic of exhausted T cells. Thus, this phenotype identified T cells that were truly exhausted and correlates well with previously established paradigm that co-expression of TIM3 with other inhibitory receptors such as PD1 contributes to impairment of T cell function during chronic inflammatory conditions. Most importantly, treatment of chronically infected mice with blocking antibodies specific for TIM3 led to a significant gain in bacterial control. Treatment was associated with an increase in IL-2, IFNγ, and TNF production by T cells. Based on this key result, we infer that TIM3-mediated T cell exhaustion impairs host resistance to *M*. *tuberculosis*. Thus, these data suggest that blockade of inhibitory T cell signals has the potential to be a therapeutic strategy against tuberculosis. The data in our report significantly advances our current knowledge of the biology of TIM3, the role of inhibitory T cell receptors during chronic infection, and the pathogenesis of tuberculosis.

## Introduction

In countries where tuberculosis exists with a low prevalence, T cell immunity to *Mycobacterium tuberculosis* enforces latency in 90% of infected people and prevents the development of clinical disease. However, in countries with endemic tuberculosis the cumulative risk for developing active tuberculosis increases with multiple exposures [1]. We hypothesize that chronic antigen stimulation from persistent subclinical infection could induce T cell exhaustion and contribute to the pathogenesis of tuberculosis. T cell exhaustion develops as a step-wise loss of proliferation, cytokine production, and CTL activity during chronic infection caused by HIV, HCV, and HBV; or during cancer [2, 3]. Although the transcriptional signature of dysfunctional CD4^+^ T cells obtained during chronic viral infection is distinct from that expressed by exhausted CD8^+^ T cells, exhausted CD4^+^ and CD8^+^ T cells also share certain hallmarks that are unique to T cell dysfunction [4]. Specific inhibitory receptors are induced on T cells, which transmit negative signals when they bind ligand. PD1, TIM3, LAG-3, CTLA-4, 2B4, and CD160 are all inhibitory receptors associated with T cell exhaustion [5, 6]. As negative regulators of T cell activity, these molecules prevent over-exuberant inflammation and tissue damage. However, inappropriate inhibitory signaling in tumor-infiltrating lymphocytes during cancer impairs tumor immunity. Importantly, therapeutic blockade of CTLA-4, PD1 or TIM3 reverses T cell exhaustion, improves anti-tumor T cell responses, diminishes tumor size, and increases survival [7–9].

The interaction between murine TIM3 and its ligand galectin-9 (Gal9) inhibits T cell proliferation and cytokine secretion in vitro and in vivo in murine models of multiple sclerosis [10, 11]. TIM3 expression is associated with CD8^+^ T cell exhaustion during HIV, HCV and HBV infection. Conversely, TIM3 blockade, in vivo during murine LCMV infection or ex vivo during human HIV and HCV infection, improves T cell proliferation and effector function [12–16]. While TIM3 is expressed by both CD4^+^ and CD8^+^ T cells in *M*. *tuberculosis* infected mice and in people with tuberculosis, conflicting data exists for its role during tuberculosis [17–19]. Given the important role of TIM3 in mediating T cell exhaustion during chronic viral infections, we determined whether TIM3 regulates T cell function during tuberculosis.

We show that CD4^+^ and CD8^+^ T cells become functionally exhausted during *M*. *tuberculosis* infection. Furthermore, we identify exhausted T cells as ones that express multiple inhibitory receptors including TIM3, PD1, Lag-3 and 2B4; and lose IL-2 and TNF production, and acquire IL-10 expression. Importantly, treatment of chronically infected mice with blocking antibodies to TIM3 improves T cell function and enhances bacterial clearance.

## Results

### T cell exhaustion develops during *M*. *tuberculosis* infection

**Fig 1:**
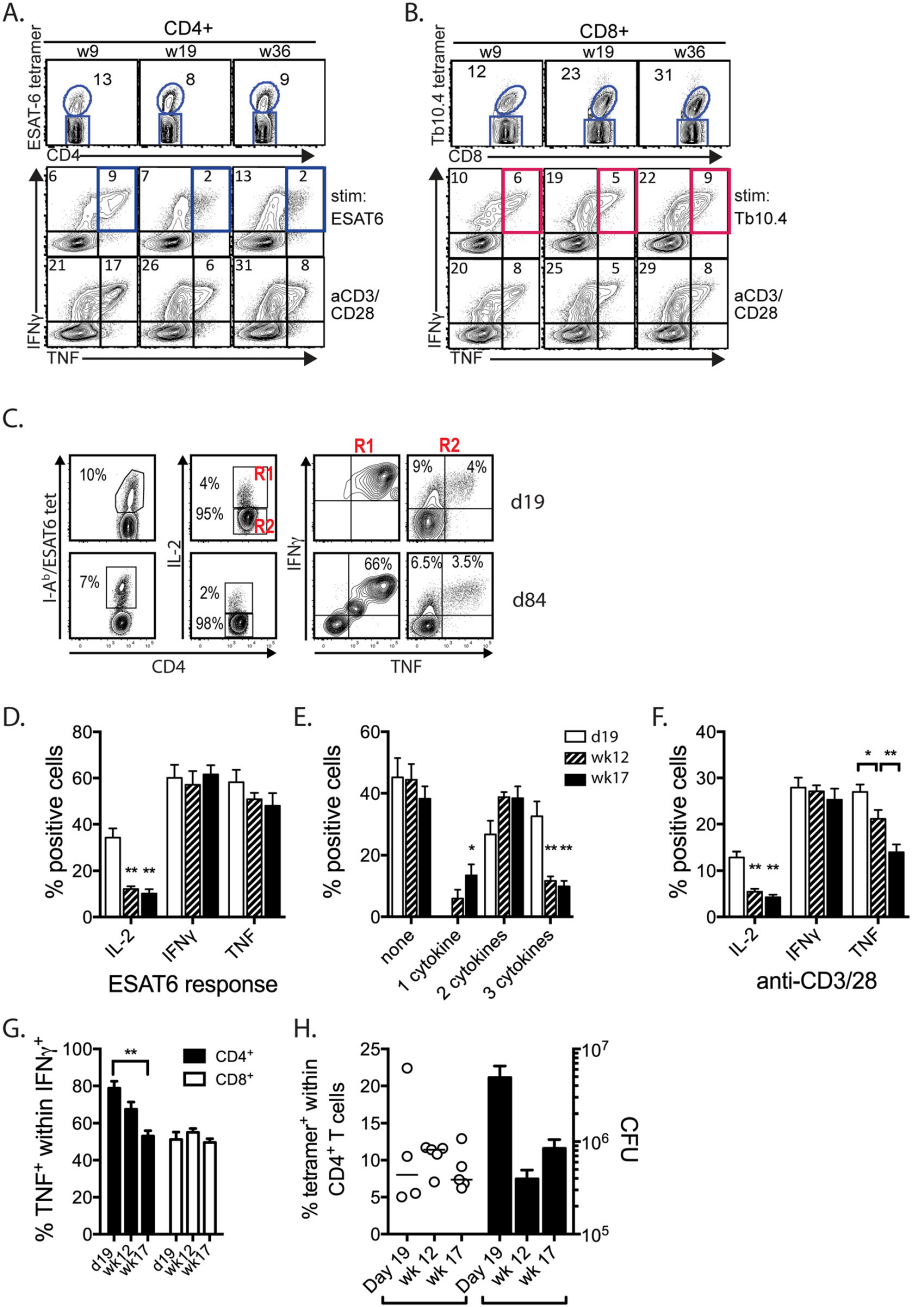
Cytokine expression in antigen-specific CD4^+^ and CD8^+^ T cells is diminished following chronic *M*. *tuberculosis* infection. (A) Representative flow cytometry data showing the frequency of ESAT6-tetramer^+^ CD4^+^ T cells after *M*. *tuberculosis* infection. At each time point, lung cells were stimulated in vitro with the ESAT6_1-15_ peptide or anti-CD3/CD28 mAbs to measure IFNγ and TNF expression. (B) The frequency of TB10.4-tetramer^+^ CD8^+^ T cells after *M*. *tuberculosis* infection. At each of time point, lung cells were stimulated in vitro with the TB10.4_4−11_ peptide or anti-CD3/CD28 mAbs and IFNγ and TNF production was measured. (C) Representative flow cytometry data of ESAT6-tetramer CD4^+^ T cells at d19 or d84 post infection. IL-2, IFNγ, and TNF production after stimulation with ESAT6_1-15_ peptide. (D) The fraction of ESAT-specific CD4^+^ T cells that make IL-2, IFNγ, and TNF on d19 (unfilled), w12 (striped), or w17 (filled) post infection. (E) The fraction of the number of cytokines being produced by ESAT6-specefic CD4^+^ T cells. (F) The fraction of CD4^+^ T cells producing IL-2, IFNγ, and TNF on d19 (unfilled), w12 (striped), or w17 (filled) post infection. (G) The percentage of IFNγ-producing CD4^+^ and CD8^+^ T cells that also make TNF over the course of infection. (H) The fraction of ESAT6-specific CD4^+^ T cells and bacterial burden in the lungs as d19, w12, and w17 post infection. All data is representative of three independent experiments with at least five mice per time point. *p<0.05, **p<0.01, ***p<0.001, one-way anova compared. Bars represent mean ± SEM. The “background” cytokine production, defined as cytokine production that occurs in the absence of specific stimulation was subtracted for each sample before calculations or normalizations were performed.

We previously found that the frequency of antigen-specific T cells (based on tetramer staining) outnumbered those that produced IFNγ in vitro, raising the possibility that some T cells in the lungs of *M*. *tuberculosis* infected mice become exhausted [20]. To ascertain whether T cell exhaustion develops during *M*. *tuberculosis* infection, we stimulated CD4^+^ and CD8^+^ T cells obtained from the lungs of infected mice ex vivo and measured their IFNγ and TNF production after stimulation with the MHCII-restricted ESAT6_1-15_ or the MHCI-restricted TB10.4_4−11_ peptide epitopes. In some of these experiments, tetramer staining to enumerate ESAT6-specific CD4^+^ T cells and TB10.4-specific CD8^+^ T cells was performed in parallel (Fig 1a and 1b). Over the course of infection, the nature of the cytokine response changed within the T cell compartment. The ratio of IFNγ^+^TNF^+^ (e.g., double producers) to total IFNγ producers fell as infection persisted (Fig 1a and 1b). This measurement is independent of the number of responding cells, and was consistent with the development of T cell exhaustion (Fig 1a and 1b). The loss of TNF occurred despite a constant, or even increasing number, of antigen-specific T cells, based on tetramer staining (Fig 1a and 1b). Similar results were obtained following brief activation of T cells with anti-CD3/CD28 antibodies (Fig 1a and 1b, indicating that this was a general property of T cells recruited to the lungs of *M*. *tuberculosis* infected mice. While these results suggested the development of T cell exhaustion based on the during chronic *M*. *tuberculosis* infection, the data from three similar experiments were not consistent (S1 Fig). The source of the variability was not immediately apparent, but possibilities included the bacterial burden and for some of the late time points, survivor bias. Nevertheless, on average, there seemed to be a loss of function among CD4^+^ T cells, although the magnitude of the effect varied (S1 Fig).

We next looked in more detail at the ESAT6-specific CD4^+^ T cell response. CD4^+^ T cells obtained from the lungs of mice 18–20 days after infection were stimulated with ESAT6 peptide or anti-CD3/28 mAb. Importantly, we carefully subtracted the background (based on unstimulated conditions) and in the case of the ESAT6-specific response, normalized the cytokine responses to the frequency of tetramer^+^ CD4^+^ T cells. Early after infection, many of the ESAT6-specific CD4^+^ T cells were polyfunctional based on their production of IL-2, IFNγ, and TNF (Fig 1c, 1d, 1e and S2 Fig). At later time points, IL-2 production was lost, which was accompanied by a proportional loss of triple cytokine producers (Fig 1d and 1e). Among the polyclonally stimulated CD4^+^ T cells, not only was IL-2 production loss, but there was also a gradual reduction in the capacity to produce TNF (Fig 1e). A decline in the ratio of IFNγ^+^TNF^+^ (e.g., double producers) to total IFNγ producers fell among polyclonally stimulated CD4^+^ T cells but not CD8^+^ T cells (Fig 1f). Importantly, these changes in the CD4+ T cell compartment occurred despite a similar frequency of ESAT6-specific T cells and an increasing bacterial burden (Fig 1h). The loss of IL-2 and TNF production and polyfunctionality is indicative of T cell dysfunction and raises the possibility that T cell exhaustion is developing during the chronic phase of tuberculosis infection.

### T cells express multiple inhibitory receptors during *M*. *tuberculosis* infection

**Fig 2:**
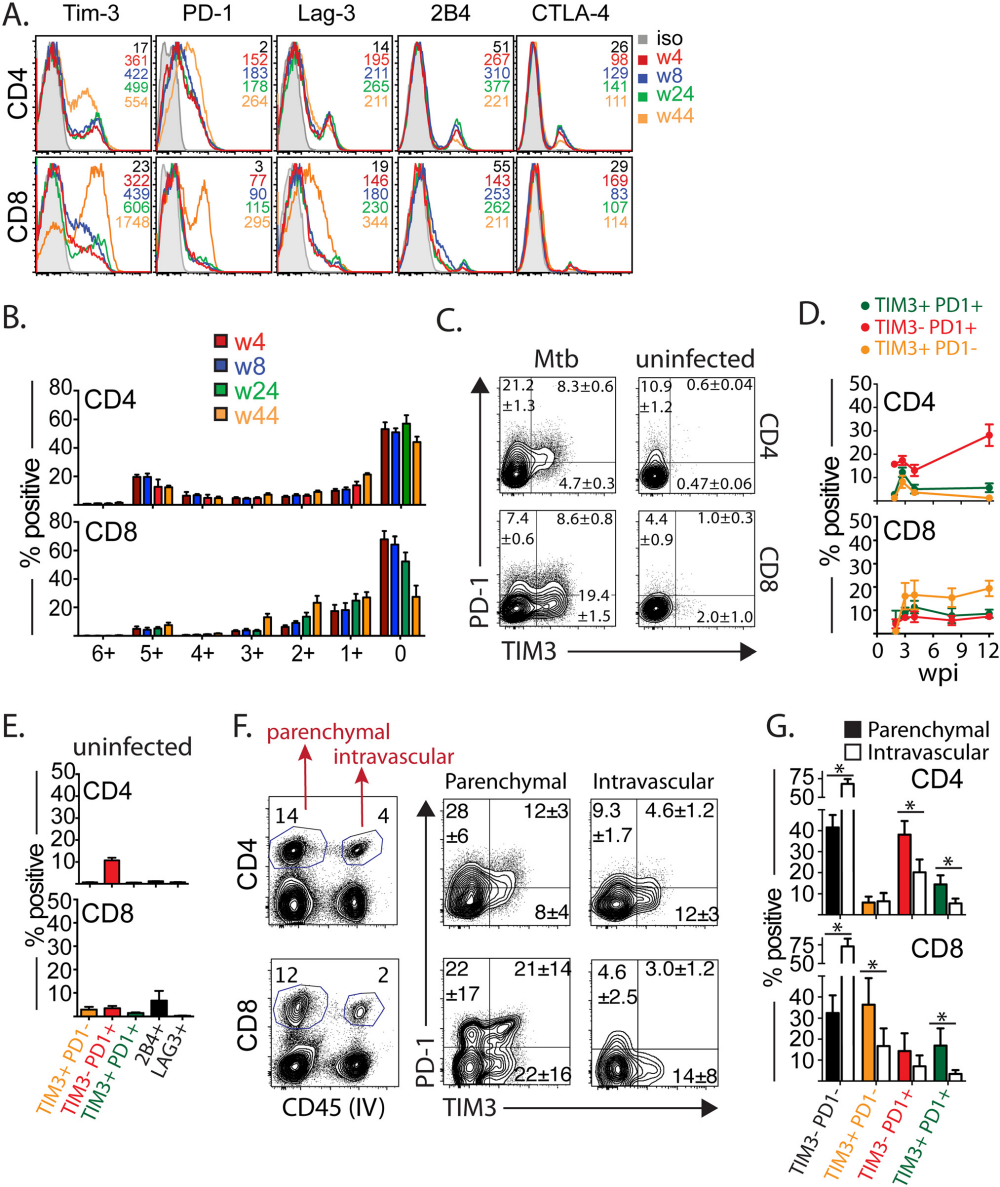
TIM3^+^ T cells are enriched for exhaustion and effector signature. (A) Expression of inhibitory receptors (TIM3, PD1, Lag-3, 2B4, and CTLA-4) by CD4^+^ and CD8^+^ T cells at week 4, 8, 24, or 44 post-*M*. *tuberculosis* infection. (B) Individual populations of CD4^+^ and CD8^+^ T cells grouped according to total number of inhibitory receptors expressed. Data was generated by Boolean gating analysis of the simultaneous expression of multiple inhibitory receptors (TIM3, PD1, Lag-3, 2B4, CTLA-4 and CD160) on CD4^+^ and CD8^+^ T cells from lungs of *M*. *tuberculosis* infected mice at weeks 4, 8, 24 and 44 after infection. (C) Expression of PD1 and TIM3 on gated pulmonary CD4 and CD8 T cells in *M*. *tuberculosis* infected (12 wpi) mice and age-matched uninfected mice. Representative FACS plots shown for one out of 5–6 mice per group (D) Frequency of CD4^+^ or CD8^+^ T cells that express TIM3 or PD1 at different time points after *M*. *tuberculosis* infection. (E) Frequency of CD4^+^ or CD8^+^ T cells that express TIM3, PD1, 2B4, or LAG-3 in uninfected mice (F) Left panel, representative FACS plots of CD4^+^ and CD8^+^ T cells at 43 wpi that stained for CD45 antibody injected intravenously. Right panel, frequency of TIM3 and PD1 within CD45^+^ (intravascular) or CD45^-^ (parenchymal) CD4^+^ and CD8^+^ T cells. (G) Frequency of CD4^+^ or CD8^+^ T cells that express TIM3 or PD1 in the parenchymal or intravascular space after *M*. *tuberculosis* infection. Data is representative of 4 (A, B) or 3 (C-E, F, G) independent experiments, each involving 5–8 mice per time point and per group. *p<0.05, by one-way anova compared. Bars represent mean ± SEM.

While there has been a systematic evaluation of the expression of canonical inhibitory receptors such as TIM3, PD1, LAG-3 among others in chronic LCMV infection, knowledge about the longitudinal expression of these molecules following *M*. *tuberculosis* infection is sparse. To evaluate whether the loss of T cell function (Fig 1) is associated with gain of cell-surface inhibitory receptors, we next measured the expression of six inhibitory receptors by CD4^+^ and CD8^+^ T cells in the lungs of *M*. *tuberculosis* infected mice (Fig 2a). TIM3, PD1, and LAG-3 were expressed on a substantial number of CD4^+^ and CD8^+^ T cells and their expression increased significantly late during infection (measured through week 44, Fig 2a). In contrast, 2B4 and CTLA4 were expressed by a small population of CD4^+^ T cells, and very little expression is seen in CD8^+^ T cells.

Up to 30% of pulmonary CD4^+^ express three or more inhibitory receptors, and at some time points, nearly 20% expressed five inhibitory receptors (Fig 2b). In contrast, <10% of CD8^+^ T cells express three or more inhibitory receptors suggesting that inhibitory receptors are regulated differently on CD4^+^ and CD8^+^ T cells (Fig 2b). We next focused on the expression of TIM3 and PD1, the two most abundantly expressed inhibitory receptors (Fig 2a). TIM3 expression is bimodal, with distinct populations of T cells expressing or lacking TIM3 (Fig 2c). In contrast, PD1 expression was more variable, with a continuum of low to high expression. We also noted significant mouse-to-mouse variability. There were fluctuations in the expression of TIM3 and PD1, particularly in the CD4^+^ T cell compartment coinciding with the initiation of adaptive immunity (Fig 2d). Subsequently, TIM3 and PD1 expression by CD4^+^ and CD8^+^ T cells increased late during *M*. *tuberculosis* infection compared to age-matched uninfected mice (Fig 2c right panels, Fig 2e). These results show that PD1 was the dominant inhibitory receptor expressed by CD4^+^ T cells. CD4^+^ T cells also expressed TIM3 were also detected, mostly as TIM3^+^PD1^+^ cells and smaller populations of TIM3^+^PD1^–^ cells. In contrast, TIM3 was the dominant inhibitory receptor expressed by CD8^+^ T cells. Among CD8^+^ T cells, TIM3^+^PD1^–^ cells were most frequent, with smaller populations of TIM3^+^PD1^+^ or TIM3^–^PD1^+^ T cells (Fig 2c and 2d).

Expression of distinct inhibitory receptors, such as LAG-3, PD1 and 2B4 are often coregulated, and coexpression of these inhibitory receptors is associated with greater T cell exhaustion and more severe LCMV infection [21]. To confirm whether TIM3^+^PD1^+^ T cells coexpress other inhibitory receptors, we measured expression of LAG-3, 2B4, CD160, and CTLA-4 within TIM3 and PD1 expressing CD4^+^ and CD8^+^ T cell populations (S3 Fig). Interestingly, 80% of CD4^+^ T cells and 60% of CD8^+^ T cells identified as TIM3^+^PD1^+^ also co-expressed up to three other inhibitory receptors. In contrast TIM3^–^PD1^–^ T cells frequently did not co-express other inhibitory receptors, regardless of the time point analyzed (S3 Fig). Thus similar to other chronic viral infections, inhibitory receptors are often co-expressed on T cells in chronic *M*. *tuberculosis* infection.

Recently, differences between lung homing (parenchymal) CD4^+^ T cells and those that reside within the lung vasculature have been identified [22]. Given that parenchymal CD4^+^ T cells are crucial for *M*. *tuberculosis* control, it was surprising that they are more likely to express PD1 and produce less IFNγ [22]. While it is possible that PD1 is behaving as an activation marker, another hypothesis is that T cells in the lung parenchyma undergo greater chronic antigen stimulation, which induces T cell exhaustion. Therefore, we next determined whether TIM3 and PD1 expression by T cells in the two different compartments differs. Towards this end, we used the well-established strategy of injecting labeled anti-CD45 antibody intravenously just before euthanasia to identify cells in the vasculature versus cells in the lung parenchyma [22, 23]. Consistent with the results of Sakai et al [22], T cells in the “parenchymal” compartment more frequently expressed PD1 (Fig 2f and 2g) and the levels were higher compared to T cells in the intravascular space. Similarly, In contrast, TIM3 was expressed by more T cells in the parenchymal compartment, particularly among CD8^+^ T cells. As above, PD1 was the dominant inhibitory receptor expressed by parenchymal CD4^+^ T cells, although TIM3^+^PD1^+^ CD4^+^ T cells were also detected (Fig 2f and 2g). PD1 and TIM3 expression among CD8^+^ T cells was bimodal, with significant TIM3/PD1 co-expressing population detected within the lung parenchymal population (Fig 2f and 2g). Thus, these data indicate that T cells in lungs of *M*. *tuberculosis* infected mice express multiple inhibitory receptors and distinct subsets of CD4^+^ and CD8^+^ T cells coexpress TIM3 and PD1.

### T cells that coexpress TIM3 and PD1 are dysfunctional

**Fig 3:**
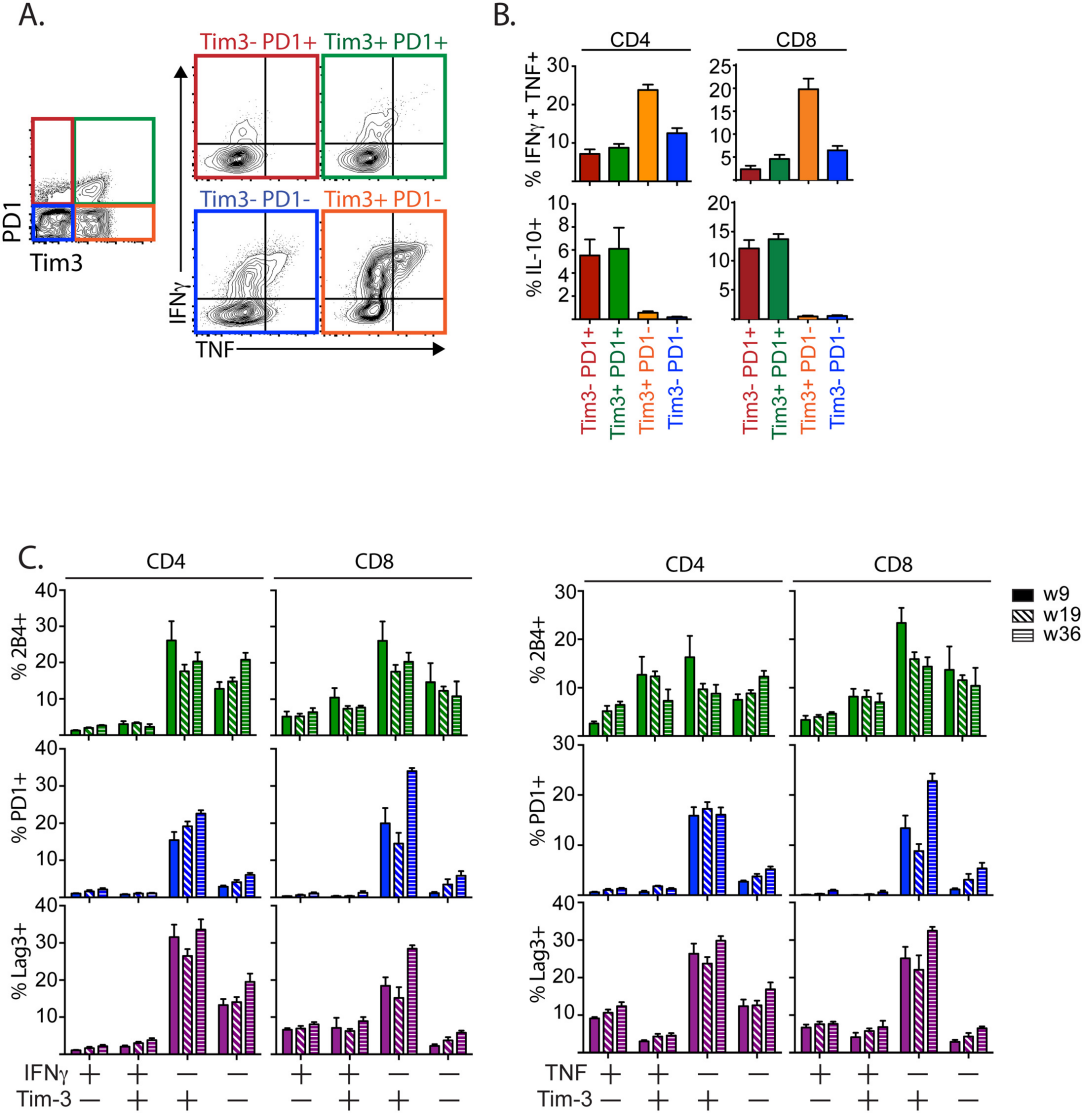
Distinct TIM3-expressing T cells display discrete functions. (A) CD4^+^ or CD8^+^ T cells from the lungs of mice 45 weeks after *M*. *tuberculosis* infection were stimulated with anti-CD3/CD28 mAbs in vitro and their expression of TIM3 and PD1, and production of IFNγ and TNF analyzed by flow cytometry. Representative gating showing the cytokine production by each of the TIM3/PD1-expressing T cell populations. (B) Frequency of TIM3/PD1-expressing CD4^+^ or CD8^+^ T cells that produce IFNγ^+^TNF^+^ or IL-10. (C) Expression of the inhibitory receptors PD1, LAG-3 or 2B4 by TIM3-expressing T cells that produce IFNγ or TNF. Data in A, B and C is representative of 3 independent experiments with 5–8 mice per time point per experiment. Bars represent mean ± SEM.

The loss of cytokine production (Fig 1) and their expression of TIM3 and PD1 (Fig 2) by T cells in the lung parenchyma of *M*. *tuberculosis* infected mice suggested that these T cells may be exhausted. To determine whether TIM3^+^PD1^+^, Tim3^–^PD1^+^ and TIM3^+^PD1^–^ T cells differ functionally, we measured cytokine production at 45 weeks post *M*. *tuberculosis* infection. After anti-CD3/CD28 mAb stimulation, TIM3^+^PD1^–^ T cells produced more IFNγ and TNF than other T cells even late during infection (Fig 3a and 3b). In contrast, TIM3^+^PD1^+^ CD4^+^ and CD8^+^ T cells produced less IFNγ or TNF, and expressed more IL-10 compared to the TIM3^+^PD1^-^ population, indicating they are likely to be dysfunctional (Fig 3a, 3b and S4 Fig [24]). We next determined the expression of other inhibitory receptors by cytokine producing T cells. TIM3^+^ CD4^+^ and CD8^+^ T cells that had diminished IFNγ and TNF responses expressed significantly more PD1, Lag-3 and 2B4, at several time points during chronic infection (Fig 3c). Thus, PD1, Lag-3 and 2B4 co-expression by TIM3^+^CD4^+^ or TIM3^+^CD8^+^ T cells identifies T cells that are unable to produce optimal IFNγ. Therefore, TIM3^+^ T cells that coexpress multiple inhibitory receptors (e.g., TIM3^+^PD1^+^) mark T cells that are more likely to be exhausted.

**Fig 4:**
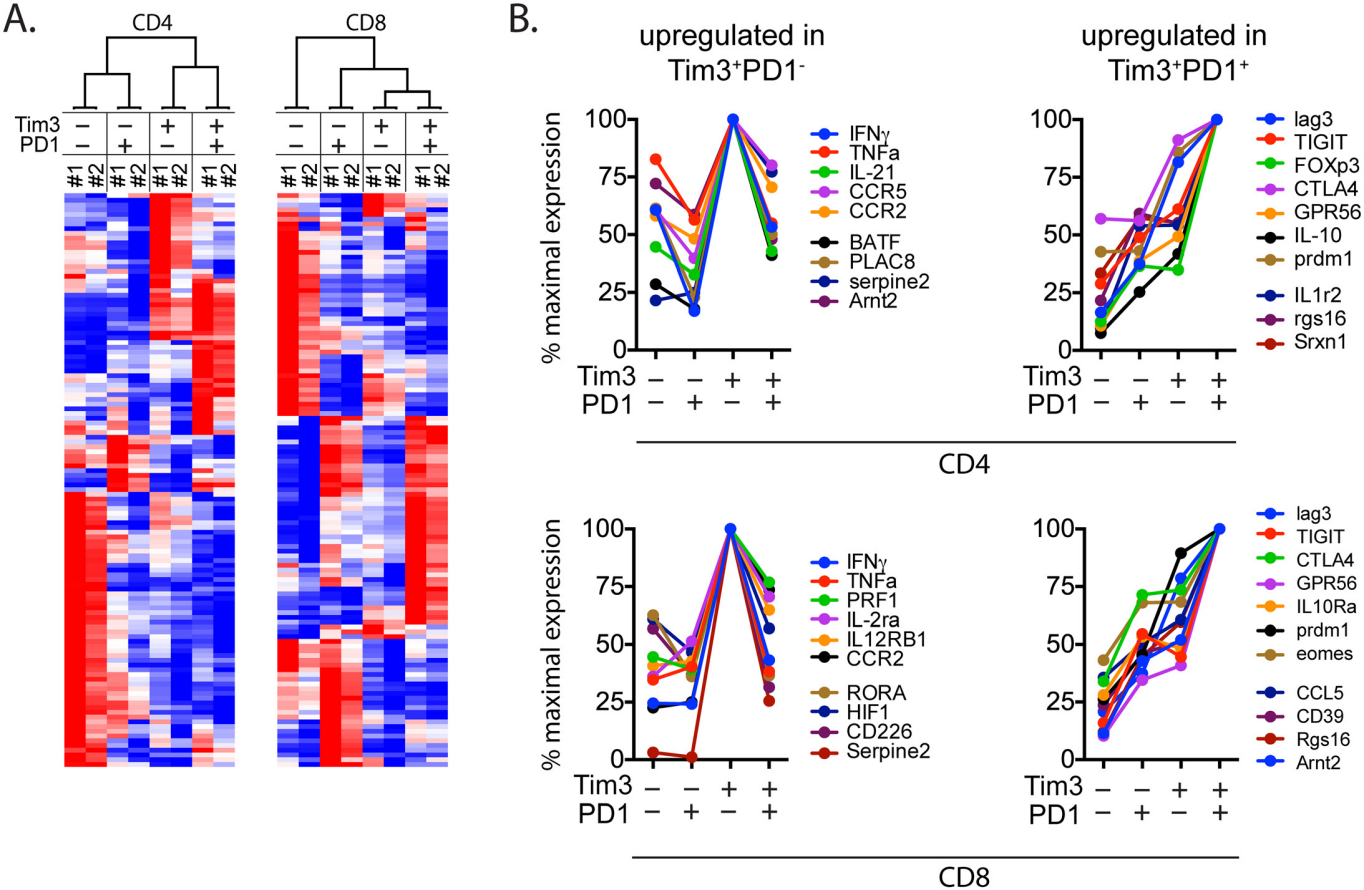
Two distinct subsets of TIM3 expressing T cells exhibit divergent molecular signatures. (A) CD4^+^ and CD8^+^ T cell populations sorted based on their TIM3 and PD1 expression was subjected to nanostring codeset and gene expression analyses. Heatmap of differentially expressed genes by TIM3^–^PD1^–^, TIM3^–^PD1^+^, TIM3^+^PD1^–^ and TIM3^+^PD1^+^ CD4^+^ or CD8^+^ populations are shown. Blue indicates low relative expression and red, high relative expression. #1 and #2 indicate data from two independent experiments. (B) Fold expression of genes were normalized with respect to highest value among the four TIM3/PD1-expressing populations. Value of 100 indicates preferential expression of a gene set to a particular TIM3/PD1 expressing population and allows assessing population-specific gene patterns. Data is representative of 2 independent experiments.

To determine whether TIM3^+^PD1^+^ T cells are functionally exhausted and exhibit a molecular signature distinct from TIM3^+^PD1^–^ and TIM3^–^PD1^+^ populations in vivo, we sorted four populations of CD4^+^ or CD8^+^ T cells from the lungs of *M*. *tuberculosis* infected mice: a) TIM3^+^PD1^–^; b) TIM3^+^PD1^+^; c) TIM3^–^PD1^+^; and d) TIM3^–^PD1^–^; and analyzed them by Nanostring without further activation (gating shown in S5 Fig). We identified specific transcriptional signatures associated with TIM3 or PD1-expressing CD4^+^ and CD8^+^ T cells (Fig 4a, S1 Table). Inhibitory molecules including Lag-3, TIGIT, CTLA4, IL-10 and the transcription factor, Blimp1 (*prdm1*), all associated with T cell exhaustion, were more highly expressed in TIM3^+^PD1^+^ T cells than in other populations (Fig 4b). Interestingly, TIM3^+^PD1^–^ T cells expressed higher levels of genes typically associated with T cell effector function. For example, TIM3^+^PD1^–^ CD4^+^ T cells expressed higher levels of pro-inflammatory cytokines associated with anti-bacterial effector function such as IFNγ and TNF, and chemokine receptors CCR2 and CCR5 (Fig 4b). TIM3^+^PD1^–^ CD8^+^ expressed more IFNγ, TNF, and perforin than TIM3^+^PD1^+^ CD8^+^ T cells (Fig 4b). To further establish that TIM3^+^PD1^–^ vs. TIM3^+^PD1^+^ are functionally distinct populations, we identified 29 genes previously determined to be associated with T cell exhaustion in CD8^+^ T cells following chronic LCMV infection and are represented in our Nanostring code set [see methods, [25]]. Twenty-two trended similarly as the exhaustion signature (up or down regulated accordingly, Chi square two-tailed p = 0.0053), with 15 genes showing a fold change of 1.5 and above. Comparing the variance of the expression values for this subset of 22 genes over all conditions to random sets of the same size shows a significant increase for this specific subset (Welch Two Sample t-test, p-value = 0.04498), showing that these specific conditions have a significant effect on this specific subset, TIM3^+^PD1^+^ population.

Thus, transcriptional signature correlate with protein expression levels for cytokines and other inhibitory receptors (Figs 3 and 4), strongly indicating that TIM3^+^PD1^–^ T cells still have the capacity to produce cytokines, while TIM3^+^PD1^+^ T cells appear to be functionally exhausted in lungs of mice with chronic *M*. *tuberculosis* infection.

### TIM3 impairs clearance of *M*. *tuberculosis*

**Fig 5:**
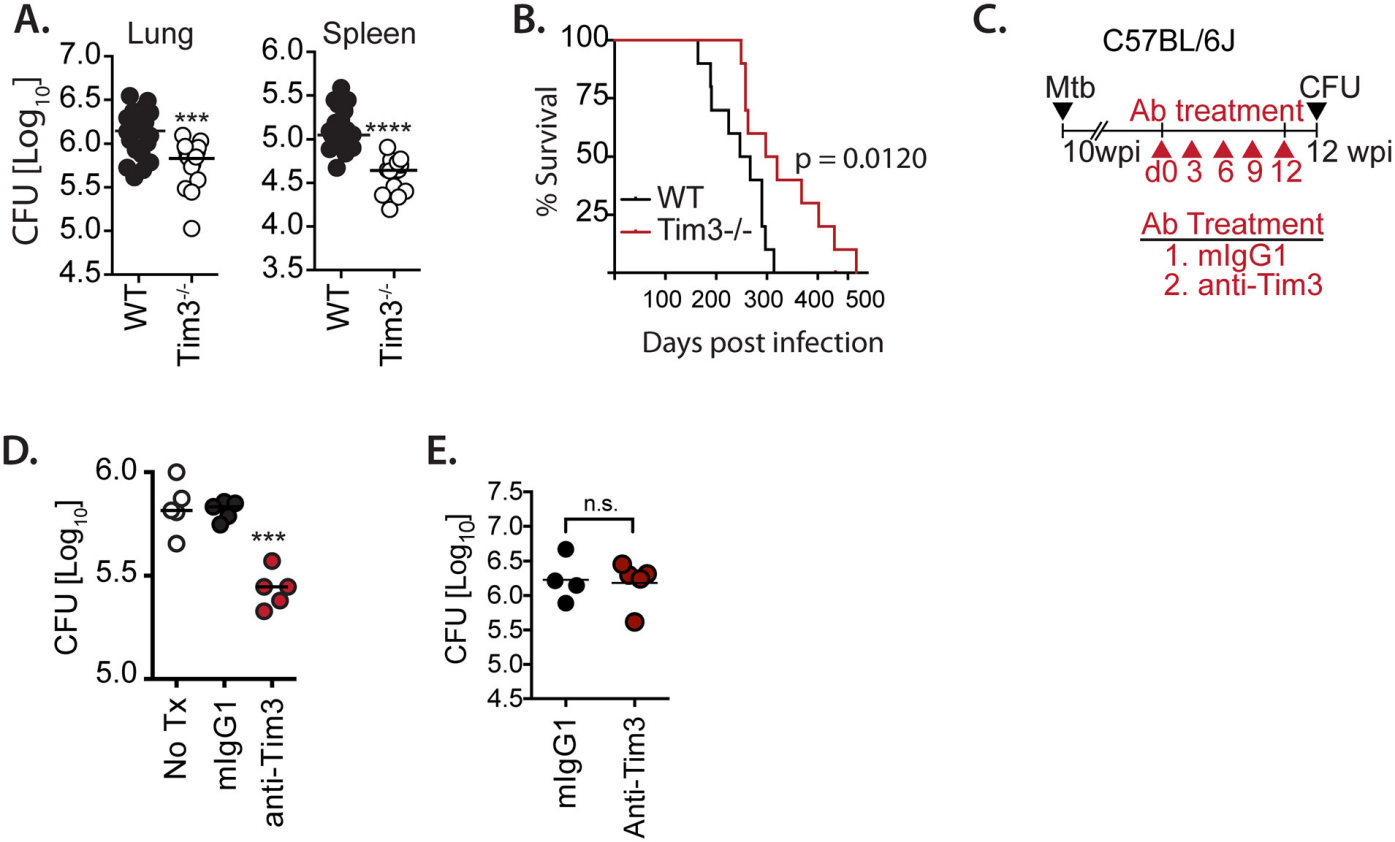
TIM3 impairs clearance of *M*. *tuberculosis*. (A) Bacterial load in the lungs and spleens of WT BALB/c and TIM3^-/-^ mice at wk4 post-infection. (B) Percent survival in *M*. *tuberculosis* infected WT and TIM3^-/-^ mice. (C) Therapeutic protocol for TIM3 blockade in C57BL/6J mice. Beginning at week 10 post-infection, chronically infected mice were treated every third day for two weeks with isotype-matched control antibody or anti-TIM3 mAb. (D) Bacterial load in the lungs of C57BL/6J mice treated with isotype-matched control antibody or anti-TIM3 mAb. (E) Bacterial load in the lungs of TCRα^-/-^ mice treated with murine IgG1 control antibody (mIgG1) or anti-TIM3 mAb. No Tx, No treatment. Data is representative of 5 (A), 1 (B), 2 (D), independent experiments. *p<0.05, **p<0.01, ***p<0.001, ****p<0.0001 for: (A) student’s t-test; (B) Log-rank (Mantel-Cox) test; (D) one-way Anova with Dunnett’s post-test. Bars represent mean ± SEM.

To determine how TIM3 affects host resistance, WT and TIM3^-/-^ mice were infected with *M*. *tuberculosis*. WT mice had more bacteria in their lungs compared to TIM3^-/-^ mice after 28 days and at later time points (Fig 5a). WT mice succumbed to *M*. *tuberculosis* infection earlier than TIM3^-/-^ mice (median survival 309 vs. 257 days, p<0.01) (Fig 5b). Thus, TIM3 expression led to a moderate impairment in control of *M*. *tuberculosis* infection.

Blockade of inhibitory receptor/ligand interaction reverses T cell exhaustion in vitro and in vivo, an effect termed ‘reinvigoration’ [13, 26]. To further clarify whether some of the TIM3-expressing T cells were exhausted and possibly impaired T cell mediated control of *M*. *tuberculosis* infection, we sought to determine whether TIM3 blockade could improve bacterial control in infected mice. Based on the kinetics of TIM3 expression and T cell dysfunction, significant T cell exhaustion did not develop until 10–12 weeks after infection (Figs 1 and 2). Therefore, ten weeks after infection, C57BL/6J mice were treated with anti-TIM3 mAb or mIgG1 control antibody for two weeks (Fig 5c). TIM3 blockade led to a significant reduction in pulmonary CFU in C57BL/6 mice (Fig 5d). Although we assume that the effect of 5D12 antibody treatment is primarily mediated by blocking TIM3 expressed by T cells, TIM3 is also expressed by alveolar macrophages, raising the possibility that the 5D12 antibody is having a direct effect on TIM3^+^ macrophages to promote anti-bacterial activity.

To address this possibility, we first confirmed that TIM3 is expressed by lung myeloid cells in uninfected and *M*. *tuberculosis* infected mice. TIM3 expression was detected on alveolar macrophages and other macrophages from the lungs of uninfected mice, but not DC, as reported [27]. Myeloid cells from the lungs of infected mice also expressed TIM3, but at reduced levels (S6 Fig). Previous work has shown that TLR agonists such as LPS and R848 downregulate cell surface TIM3 on peripheral monocytes in healthy human donors [28]. The lower TIM3 expression by lung infiltrating myeloid cells could possibly result from exposure to TLR ligands expressed by *M*. *tuberculosis*.

Given the expression of TIM3 on cells that could potentially be infected by *M*. *tuberculosis*, we next considered whether the 5D12 mAb directly activated macrophages to express antibacterial activity. TCRα^-/-^ mice were exposed to aerosolized *M*. *tuberculosis* and then treated with 5D12 anti-TIM3 mAb or mIgG1 control for two weeks. TIM3 blockade had no effect on the course of *M*. *tuberculosis* infection in TCRα^-/-^ mice and the lung bacterial burden was similar in mice treated with anti-TIM3 and mIgG1 (Fig 5e). Thus, 5D12 anti-TIM3 mAb is not working in a T cell independent manner (e.g., directly activating myeloid cells). Based on these data, we surmise that the net action of TIM3 as a negative regulator of T cell immunity is a quantitatively important effect.

### TIM3 blockade improves T cell function and disease outcome in susceptible mice

**Fig 6:**
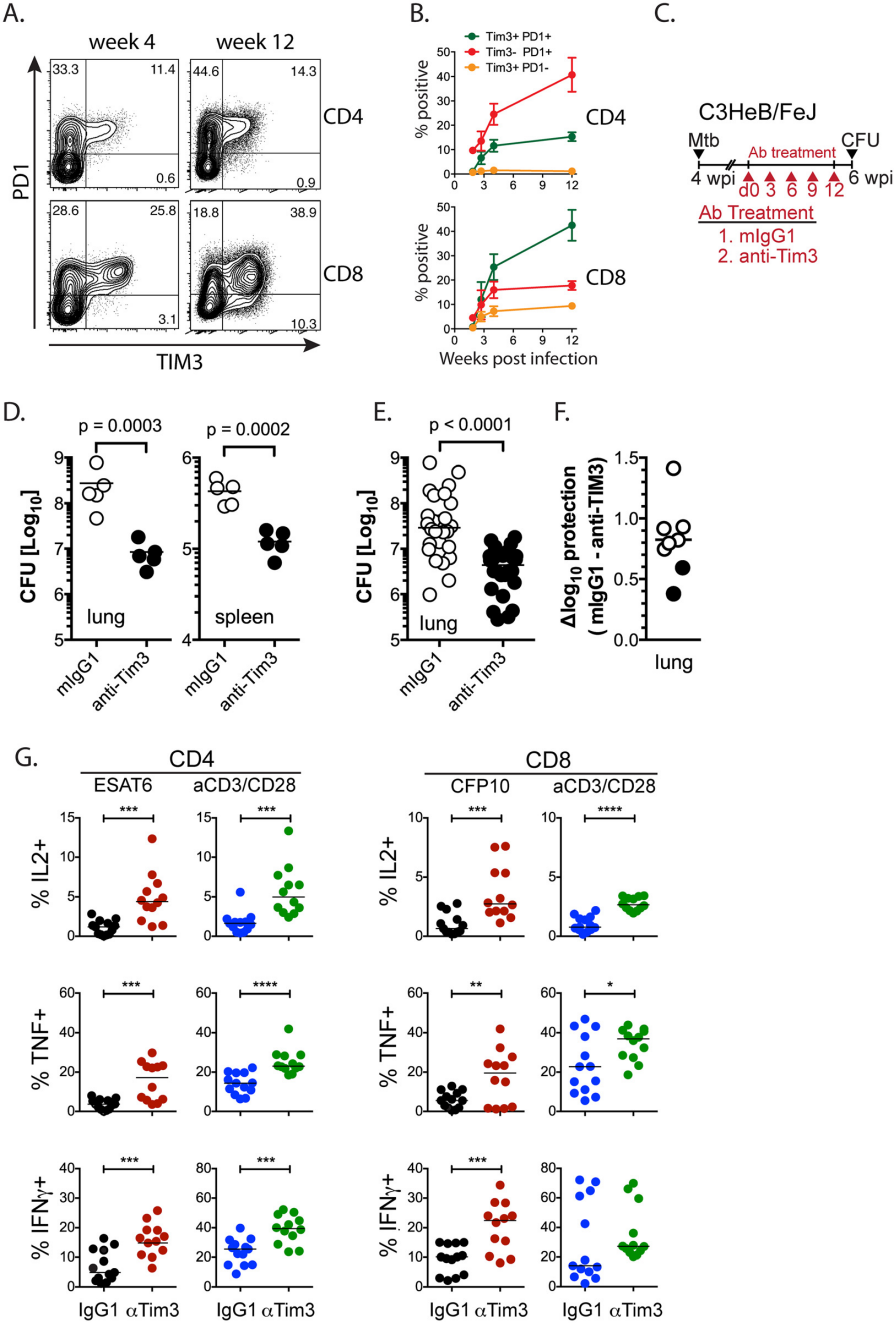
TIM3 blockade improves T cell function and disease outcome. (A) Representative contour plots for PD1 and TIM3 expression on pulmonary CD4^+^ and CD8^+^ T cells in susceptible C3HeB/FeJ mice, 4 and 12 weeks after *M*. *tuberculosis* infection. (B) Emergence of TIM3^+^PD1^+^, TIM3^+^PD1^-^ and TIM3^-^PD1^+^ CD4^+^ and CD8^+^ T cells populations in C3HeB/FeJ mice following *M*. *tuberculosis* infection. Frequency of CD4^+^ or CD8^+^ T cells that are positive or negative for TIM3 and PD1 expression at different times post *M*. *tuberculosis* infection is plotted. Each point represents the mean ± SEM of 5 mice per strain per time point, and is representative of 2–3 independent experiments. (C) Protocol for TIM3 blockade in C3HeB/FeJ mice. C3HeB/FeJ mice were treated every third day for two weeks with isotype-matched control antibody or anti-TIM3 mAb, starting 4 weeks after *M*. *tuberculosis* infection. (D) Data from a representative experiment shows the bacterial loads in lung and spleen. (E) Cumulative results from all blocking experiments performed in C3HeB/FeJ mice representing 26 mice/group from six independent experiments. Each point represents lung CFU from an individual mouse. p<0.0001 by unpaired t-test after log_10_ transformation. (F) The Δlog_10_ protection [control CFU—treatment CFU] from eight independent experiments. Black circles, C57BL/6 experiments; white circles, C3HeB/FeJ experiments. (G) Production of IFNγ, TNF and IL-2 by CD4^+^ and CD8^+^ T cells from the lungs of infected C3HeB/FeJ mice that had been treated as described above. T cells were stimulated in vitro with ESAT6_53-71_ or CFP10_32-39_ peptides (recognized by CD4^+^ or CD8^+^ T cells, respectively) or anti-CD3/28 mAbs. Data is from 12–13 mice from three independent experiments tested by unpaired t-test: *, p<0.05; **, p<0.01; ***, p<0.001; ****, <0.0001. Bars represent median.

C57BL/6 mice are relatively resistant to tuberculosis and are able to contain *M*. *tuberculosis* infection for nearly a year before succumbing to infection. Although we cannot currently assess to what degree T cell exhaustion may contribute to their demise, we assume that T cell exhaustion develops in part because of long-term chronic antigen stimulation. We hypothesize that T cell exhaustion would develop earlier in susceptible mouse strains such as C3H mice because of their greater bacterial burden and antigen load [29–31]. Compared to C57BL/6 mice, both CD4^+^ and CD8^+^ T cells in the lungs of C3HeB/FeJ mice expressed more PD1 following low dose *M*. *tuberculosis* infection (Fig 6a). Indeed, with progression of *M*. *tuberculosis* infection, there were more TIM3^+^PD1^+^ CD8^+^ T cells than TIM3^+^PD1^-^ or TIM3^-^PD1^+^ ‘single positive’ T cells in lungs of C3HeB/FeJ, consistent with the idea that chronically elevated levels of antigen promote T cell exhaustion (Fig 6a and 6b).

We next determined whether TIM3 blockade alters host resistance in susceptible C3HeB/FeJ mice. Four weeks after infection of C3HeB/FeJ mice, treatment was initiated using an mIgG1 control mAb or anti-TIM3 mAb for two weeks (Fig 6c). As we observed for C57BL/6 mice, TIM3 blockade led to a CFU reduction in the lungs and spleens of *M*. *tuberculosis* -infected C3HeB/FeJ mice (Fig 6d and 6e). In all, TIM3 blockade was performed in eight independent experiments, twice in C57BL/6 mice (open symbols, Fig 6f) and six times in C3HeB/FeJ mice (closed symbols, Fig 6f), and we observed a statistically significant lung CFU reduction in all eight experiments.

To determine whether improved T cell function correlated with the enhanced protection observed after TIM3 blockade, T cells from C3HeB/FeJ mice treated with isotype control or anti-TIM3 mAbs were stimulated with class I MHC-restricted (CFP10_32-39_) or class II MHC-restricted (ESAT6_53-71_) peptide epitopes, or anti-CD3/CD28 antibodies. Both CD4^+^ and CD8^+^ T cells produced more IFNγ, TNF and IL-2 after TIM3 blockade (Fig 6g). These data show that T cell exhaustion impairs T cell function during chronic *M*. *tuberculosis* infection, but also therapeutic targeting of inhibitory T cell signals can reverse exhaustion and improve bacterial control.

## Discussion

TIM3 is a key negative regulator of T cell function, which has a pathological role in autoimmune diseases, chronic viral infections and malignancies [7, 9, 13, 14, 32–34]. Increased TIM3 expression is observed in people and non-human primates with active tuberculosis, and in this report, we show that TIM3 affects T cell immunity to *M*. *tuberculosis*. We find that TIM3 is expressed by two distinct subsets of T cells: 1) T cells that express TIM3 but not other inhibitory receptors (e.g., TIM3^+^PD1^–^); and 2) T cells that express TIM3 and other (often multiple) inhibitory receptors (e.g., TIM3^+^PD1^+^). TIM3^+^PD1^–^ T cells produce more IL-2, TNF, and IFNγ, than other cells and share properties with effector T cells (P.J and S.M.B, manuscript in preparation). In contrast, TIM3^+^PD1^+^ CD4^+^ and CD8^+^ T cells that emerge late during chronic *M*. *tuberculosis* infection co-express other inhibitory receptors including Lag-3, produce more inhibitory cytokines (IL-10) and less pro-inflammatory cytokines (IFNγ, TNF and IL-2), and have an in vivo molecular signature that resembles exhausted T cells.

A crucial question in TB pathogenesis is why does *M*. *tuberculosis* infection develop into disease? The majority of people infected by *M*. *tuberculosis* successfully control the infection and only a minority develop clinical disease, often despite containing the infection for years. This is also pertinent to animal models: some mouse strains control *M*. *tuberculosis* infection for up to a year without clinical symptoms but eventually succumb to disease. While impairment of cell-mediated immunity can lead to a failure of immunity, whether caused by HIV, immunosuppressive drugs, malnutrition or other causes, many people who develop TB do not have any identifiable risk factors. One possibility is that Tregs or inhibitory cytokines negatively affects host resistance [35, 36]. Here we show that T cells expressing inhibitory receptors are functionally exhausted. We do not yet know whether T cell exhaustion, and maybe inappropriate expression of inhibitory receptors, leads to immune failure and bacterial recrudescence. Alternatively, T cell exhaustion could develop secondary to bacterial recrudescence. Greater bacterial growth would increase the antigen load, and could lead to chronic antigen stimulation and T cell exhaustion. For example, C3HeB/FeJ mice, which have elevated lung CFU early during their disease course, manifest greater T cell exhaustion earlier during tuberculosis infection. While C3H mice have a genetic basis for their susceptibility [37], we propose that once recrudescence and immune failure starts to happen, T cell exhaustion can hasten the demise of an infected individual. Importantly, as shown most dramatically by our experiments with C3HeB/FeJ mice, there was a clear therapeutic effect of TIM3 blockade, even in susceptible individuals.

Most studies of chronic HIV, HBV, or HCV infection, or malignancy, find that most TIM3^+^ T cells coexpress PD1 (e.g., belong to the TIM3^+^PD1^+^ subset) or that TIM3^+^PD1^–^ T cells are similarly exhausted as TIM3^+^PD1^+^ T cells [7, 9, 13, 14, 32–34]. TIM3^+^PD1^–^ T cells have been shown to emerge in certain tumor models when Tregs that accumulate at early stages of tumor remodeling are depleted from the tumor microenvironment [38]. In our study, the TIM3^+^PD1^–^ or TIM3^+^PD1^+^ phenotype correlated with distinct T cell cytokine responses. TIM3^+^PD1^–^ T cells were more likely to co-produce IFNγ and TNF, while fewer TIM3^+^PD1^+^ T cells expressed IFNγ or TNF. We detected both increased IL-10 transcripts in CD4^+^ T cells, which also produced more IL-10 after in vitro stimulation. Although increased IL-10 expression is associated with CD4^+^ T cell exhaustion [4], we also detected increased production by CD8^+^ T cells after stimulation. The unique gene expression profiles of these different populations from the lungs of *M*. *tuberculosis* infected mice support TIM3^+^PD1^+^ and TIM3^+^PD1^–^ T cells having distinct functions and activation states.

Importantly, the functionally exhausted state of TIM3^+^PD1^+^ T cells appears to be detrimental to the outcome of *M*. *tuberculosis* infection since treatment with anti-TIM3 mAbs leads to a reduction of the bacterial burden in chronically infected C57BL/6 and C3H mice. As TIM3 expressed by T cells can induce anti-bacterial activity in macrophages via its ligand Gal9 and lead to enhanced *M*. *tuberculosis* control, we were initially concerned that targeted TIM3 blockade could have a detrimental effect on host resistance to *M*. *tuberculosis* [19, 39]. However, not only did TIM3 blockade reduce the bacterial load (Figs 5 and 6), but it also resulted in greater T cell cytokine production, a phenomenon referred to as T cell invigoration in other models [40]. It was particularly interesting that increased levels of IL-2 production were one of the most consistent changes after TIM3 blockade, as little IL-2 production is detected after the first 2–3 weeks of *M*. *tuberculosis* infection. These data show that T cell exhaustion impairs immunity to tuberculosis in vivo and demonstrate that re-invigoration of T cells by TIM3 blockade can be a therapeutic strategy.

Importantly, both TIM3^-/-^ mice and TIM3 blockade of intact mice lead to greater resistance to *M*. *tuberculosis* and none of our studies led to a worse outcome. It is not particularly surprising that we observed only a modest reduction in lung bacterial burden and increase in survival of TIM3^-/-^ mice after *M*. *tuberculosis* infection: enhanced resistance is difficult to detect in mice that are inherently resistant to TB [41]. Furthermore, TIM3 has other functions beyond inhibiting T cell function [42–50]. We have previously shown that Tim3 binding to Gal9 enhances host resistance against *M*. *tuberculosis* [19, 39]. Now, we show that TIM3 also has an inhibitory role. The net effect of these opposite effects is a slight increase in host resistance of TIM3^-/-^ mice. Finally, TIM3 is unlikely to be the only molecule that mediates T cell exhaustion given the association with PD1 and other inhibitory receptors.

Our results contrast with the genetic ablation of PD1 or PD-L1, which results in extreme susceptibility to *M*. *tuberculosis*, as severe as RAG^-/-^ or IFNγ^-/-^ mice [51, 52]. As transfer of PD1^-/-^ CD4^+^ T cells replicates the phenotype of intact PD1^-/-^ mice, and leads to increased bacterial burden and lung necrosis, it is likely that that PD1 has an important immunoregulatory role early during infection [51]. Based on the different susceptibility of PD1^-/-^ and PD-L1^-/-^ vs. TIM3^-/-^ mice to *M*. *tuberculosis* (Fig 5, [51]), it is clear that TIM3 and PD1 play distinct roles in host resistance to *M*. *tuberculosis* infection. This is in line with differing functions attributed to functional rescue of T cells following Nivolumab (anti-PD1) or Ipilimumab (anti-CTLA4) suggesting these immune check point molecules have evolved to affect T cell function through distinct pathways [47, 53].

Research on T cell exhaustion during infection has focused on chronic viral pathogens, such as HBV, HCV, and HIV, which are characterized by high antigen loads, and where inhibitory receptors including TIM3 and PD1, negatively regulate T cell function [13, 14, 32, 33]. Unlike LCMV infection, in which viral strains that induce acute or chronic infection facilitate the study of T cell function [3, 12], it has been hard to discern whether true T cell exhaustion develops during tuberculosis. We report here that T cell exhaustion develops late during *M*. *tuberculosis* infection, particularly in the CD4^+^ T cell compartment, based on the progressive but reversible loss of T cell function. Understanding the altered molecular pathways in different subsets of TIM3-expressing populations and how these populations could be selectively targeted therapeutically should be a promising approach to counter chronic infection caused by intracellular pathogens. Understanding why immunity fails in some individuals after *M*. *tuberculosis* infection is pivotal for the development of vaccines, host directed therapy, and public health measures.

## Methods

### Ethics statement

The animal studies were approved by the Institutional Animal Care and Use Committee at the University of Massachusetts Medical School (Animal Welfare Assurance no. A3306-01), using the recommendations from the Guide for the Care and Use of Laboratory Animals of the National Institutes of Health and the Office of Laboratory Animal Welfare.

### Mice and infections

Six- to eight-week old C57BL/6J, Balb/c, C3HeB/FeJ, or B6.129S2-Tcra^tm1Mom^/J were purchased from Jackson laboratories; TIM3^-/-^ mice (originally from Millennium Pharmaceuticals), were bred locally. All in vivo infections were performed using virulent *M*. *tuberculosis* (Erdman strain) by the aerosol route with ~200 CFU by an aerosol-generation device (Glas-Col). At different times post infection, mice were euthanized by carbon dioxide inhalation and lungs and spleens were aseptically removed. Organs were individually homogenized in 0.9% NaCl/0.02% Tween 80 with MiniBead Beater 8 (Biospec Products) and viable bacteria were enumerated by plating 10-fold serial dilutions of organ homogenates onto Remel 7H10 M.tb plates (R01610; Fisher Scientific). Colonies were counted after 21 d.

### In vivo blockade of the TIM3 pathway

All TIM3 blockade experiments were performed in chronically infected B6 (12 wks post-infection) and C3HeB/FeJ (4 wks post-infection). For TIM3 blockade, 500 μg of anti-TIM3 mAb (5D12; prepared in house) or moues IgG1 isotype control were injected intraperitoneally on d0 of treatment and 100 μg every 3d for 2 wk. The ability of anti–TIM3 mAb to block the TIM3 pathway was previously demonstrated [11]. TIM3 blockade in TCRα-/- mice were performed one day post infection with 500 μg of anti-TIM3 mAb (5D12) or mouse IgG21 isotype control were injected intraperitoneally on d0 of treatment and 100 μg every 3d for 2 wk. Lung CFU from anti-Tim3 and isotype treated mice was measured 21 days post infection.

### MHC tetramers, Abs, and flow cytometry

Anti-CD3 (clone 145-2C11), anti-CD4 (GK1.5), anti-CD8 (53–6.7), anti-CD19 (6D5), anti-CD62L (MEL-14), anti-CD44 (IM7), anti-CD45RB (C63-16A), anti-CD127 (A7R34), anti-TIM3 (2C12), anti-TIM3 (5D12; V. Kuchroo), anti-PD1 (29F.1A12), anti-Lag3 (C9B7W), anti-2B4 (M2B4CB6), anti-CD160 (7H1), anti-CTLA4 (UC10-4B9), anti-IFNγ (XMG1.2), anti-TNF (MP6-XT22), anti-IL2 (JES6-5H4), anti-IL-10 (JES6-16E3), anti-CD107a (1D4B), CD107b (M3/84), rat anti-mouse CD16/CD32 (Fc-Block) were purchased from biolegend. I-A^b^ ESAT-6_1–20_, K^b^ TB10.3/4_4–11_, and K^k^ CFP10_32–39_ MHC tetramers were produced by the National Institute of Allergy and Infectious Diseases Tetramer Core Facility (Emory University, Atlanta, GA). For staining with I-A^b^ ESAT-6_1–20_ tetramer, cells were incubated with tetramer at 1:200 dilution in complete media containing 10% FCS for 1 h at 37°C prior to staining with surface Abs. Cells were stained with MHC class I tetramers at 4°C for 30 min. For intracellular staining of cytokines IFNγ, TNF, IL-2 or IL-10, lung mononuclear cells from infected mice were cultured in complete media at 37°C with or without 10 μM peptide (to interrogate antigen-specific T cell responses, see Table 1) or 1 μg/ml anti-CD3/CD28 mAbs (to interrogate polyclonal T cell responses). Peptides used in this study are listed in Table 1. After 1 h, 50 μl brefeldin A (25 μg/ml; Sigma-Aldrich) was added and cells were cultured for an additional 4 h. After activation, the cells were washed and stained for extracellular and intracellular markers according to manufacturer’s instructions. Samples were acquired on an LSRII flow cytometer (Becton Dickinson, Franklin Lakes, NJ) or the MACSQuant Analyzer (Miltenyi Biotec, San Diego, CA) and analyzed with FlowJo software (Tree Star, Ashland, OR).

**Table 1:** *Mycobacterium tuberculosis* peptide epitopes used to stimulate T cells ex vivo for Intracellular cytokine staining assays.

Mouse strain	MHC restriction	Epitope name	Epitope sequence
C57BL/6J	K^b^	TB10.4_4−11_	IMYNPAM
	I-A^b^	ESAT6_1-15_	MTEQQWNFAGIEAAA
C3HeB/FeJ	K^k^	CFP10_32-39_	VESTAGSL
	MHC II	ESAT6_53-71_	GVQQKWDATATELNNALQN

### Nanostring

To assess differences between specific populations, T cells were first purified from lungs of *M*. *tuberculosis* infected mice were MACS purified and stained for cell surface markers such as CD4, CD8, TIM3 or PD1 from different strains of mice: C57BL/6J or C3HeB/FeJ. Stained T cells were then sorted into distinct populations under BSL-3 conditions. RNA was isolated from sorted populations using Qiagen RNeasy kit according to manufacturer’s instructions. The T cell exhaustion signature was constructed based on Affymetrix microarray dataset from D^b^-restricted GP33-specific CD8^+^ T cells on day 6, day 8, day 15, and day 30 following acute (Armstrong) or chronic (clone 13) LCMV infection [54]. The Marker Selection module of GeneE was used to rank genes by their signal-to-noise ratio [55]. A cutoff of 2 fold change (either up or down regulated), as well as, FDR<0.2 was applied to filter significantly differential expressed genes. Exhaustion signature was defined as a combined list of all the differential expressed genes (DE) between chronic and acute between each of the 4 time points. The number of DE genes for each time point is: day 6, 62 genes; day 8, 139 genes; day 15, 547 genes; day 30, 176 genes with a total of 879 unique DE genes. From this list of 879 viral exhaustion signature, 29 genes were also included in the Nanostring set (Fig 3c).

### Statistics

CFU data were log_10_ transformed before analysis. The Prism software program (GraphPad Software) was used to perform Student’s t tests and one-way ANOVA and Bonferroni’s multiple comparison post-test. A p-value of <0.05 was considered significant. The log-rank (Mantel-Cox) test was used for statistical analysis for survival experiments.

## Supporting Information

S1 Fig Cytokine production by T cells from *M*. *tuberculosis* infected mice. Three similar experiments (‘A’, ‘B’, ‘C’), all which show the kinetics of IFNγ and IL-2 production by pulmonary CD4^+^ and CD8^+^ T cells from *M*. *tuberculosis* infected mice. Lung mononuclear cells were prepared and stimulated with ESAT6 or TB10 peptide epitopes, or anti-CD3/38 mAbs in vitro. As described in the methods, TNF or IFNγ were detected by intracellular cytokine straining. ‘%TNF^+^ within IFNγ^+^’ refers to the ratio of TNF^+^IFNγ^+^ cells to total IFNγ^+^ cells. This ratio provides a functional assessment of the T cells, independently of the number of T cells at anytime point. Each point represents the mean +/- SEM for 3–6 mice/time point.
(PDF)

S2 Fig Cytokine production by T cells from *M*. *tuberculosis* infected mice. (A) Lung mononuclear cells were purified from the lungs of mice 18 days after *M*. *tuberculosis* infection and stimulated in vitro with ESAT6_1-15_ or TB10.4_4−11_ peptide, anti-CD3/CD28 mAb, or media (unstimulated) control. Representative FACS plots of CD4^+^ or CD8^+^ T cell stained for intracellular IL-2 and IFNγ. (B) Three similar experiments (‘A’, ‘B’, ‘C’), all which show the kinetics of IFNγ and IL-2 production by pulmonary CD4^+^ and CD8^+^ T cells from *M*. *tuberculosis* infected mice. The frequency of ESAT6-specific CD4^+^ or TB10.4-specific CD8^+^ T cells that produce IL-2 or IFNγ after stimulation in vitro with peptide epitopes and intracellular cytokine staining. (C) The fraction of CD4^+^ and CD8^+^ T cells producing different combinations IFNγ, TNF or IL-2 after in vitro stimulation with peptide epitopes or anti-CD3/CD28 mAb. Each pie slice represents the fraction of the total CD4^+^ or CD8^+^ T cell cytokine response that produces the combination of cytokines indicated in the legend.
(PDF)

S3 Fig Expression of inhibitory receptors by T cells expressing TIM3 and PD1. T cells were obtained from lungs of *M*. *tuberculosis* infected mice at various time points after infection (2, 8, 24, or 44 weeks) (n = 4–5 per group per time point). CD4^+^ and CD8^+^ T cells expressing Tim3 and/or PD1 were analyzed for their expression of other inhibitory receptors (LAG3, CTLA4, CD160, 2B4). 80% of TIM3^+^PD1^+^ CD4^+^ T cells co-expressed three other inhibitory receptors. This frequency was greater than TIM3^+^PD1^–^ (~40% of cells included 3 other inhibitory receptors) and TIM3^—^PD1^+^ CD4^+^ T cells (<20% of T cells included three other inhibitory receptors). In contrast TIM3^–^PD1^-^ T cells frequently did not express other inhibitory receptors, regardless of the time point analyzed. Data are representative of 2 independent experiments.
(PDF)

S4 Fig Detection of intracellular IL-10 production by T cells from the lungs of chronically infected mice. Representative flow cytometry plots of intracellular IL-10 production by TIM3- and PD1-expressing CD4^+^ (right panels) and CD8^+^ (left panels). T cells from the lungs of chronically *M*. *tuberculosis* infected mice were stimulated in vitro with anti-CD3/28 mAbs. An antibody specific for IL-10 (upper panels) or an isotype control (lower panels) was used for intracellular staining. Data are representative of 2 independent experiments, each with 3–4 mice per group.
(PDF)

S5 Fig Gating strategy for sorting of TIM3- and PD1-expressing CD4^+^ or CD8^+^ T cells for Nanostring analysis. Lung mononuclear cells were obtained by collagenase digest and T cells were enriched by negative selection using immunomagnetic beads. Lymphocytes were identified based on size and scatter, and after gating on singlets, CD4^+^ or CD8^+^ T cells were identified based on CD3^+^CD4^+^ or CD3^+^CD8^+^ expression. For each population of CD4^+^ or CD8^+^ T cells, four Tim3- and PD1-expressing populations were sorted: (1) Tim3^–^PD1^+^, (2) Tim3^+^PD1^+^, (3) Tim3^+^PD1^–^, (4) Tim3^–^PD1^–^. A sample of each sorted population was reanalyzed to verify the phenotype assess the purity before performing Nanostring analysis.
(PDF)

S6 Fig TIM3 expression by myeloid cells. Gating strategy for identifying myeloid population Tim3 expression. Representative flow cytometry plots from lungs of uninfected mice (A) and lungs of *M*. *tuberculosis* infected mice 21 days post infection (B). Cells of hematopoietic lineage were identified with CD45, then alveolar macrophages were gated on auto-fluorescence. Dendritic cells, recruited macrophages, and neutrophils were identified by CD11c, CD11b, and Ly6G expression. Having identified these various cell types, TIM3 expression by alveolar macrophages, dendritic cells (DC), and neutrophils was determined. TIM3 expression was quantitated as the percentage of positive cells and median fluorescent intensity (MFI).
(PDF)

S1 Table Data from Nanostring. (1) Tim3^–^PD1^+^; (2) Tim3^+^PD1^+^; (3) Tim3^+^PD1^–^; and (4) Tim3^–^PD1^–^ cells sorted from CD4^+^ or CD8^+^ T cells obtained from the lungs of chronically *M*. *tuberculosis* infected mice were analyzed by Nanostring using a 121 gene codeset. Normalized data from two independent experiments are shown.
(XLSX)
