# Supplementary figures and images for: TIM3 Mediates T Cell Exhaustion during Mycobacterium tuberculosis Infection
Source: PLoS Pathog. 2016 Mar 11;12(3):e1005490. doi: 10.1371/journal.ppat.1005490 (PMC4788425; doi:10.1371/journal.ppat.1005490)

CD4

aCD3/CD28

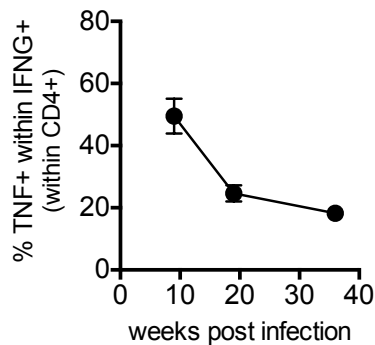

ESAT6

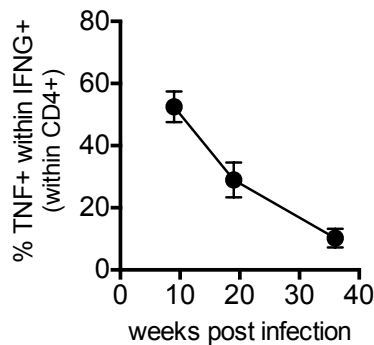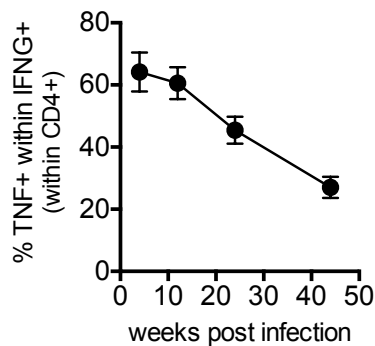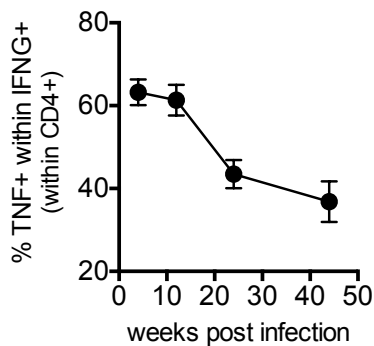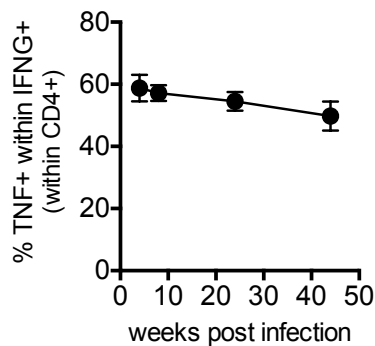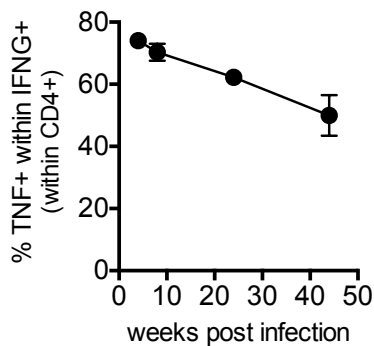

CD8

aCD3/CD28

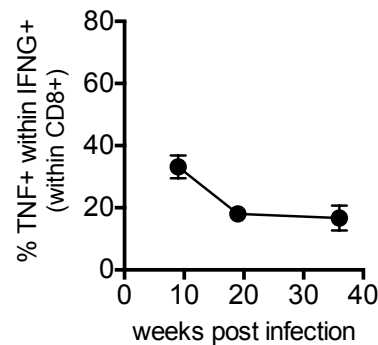TB10.4<sub>4-11</sub>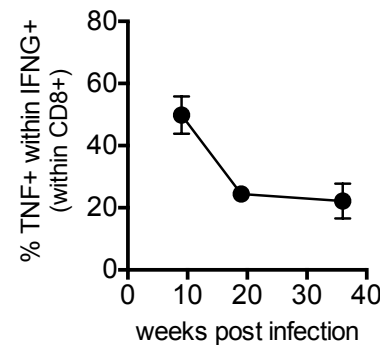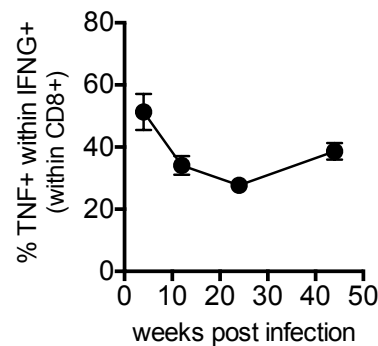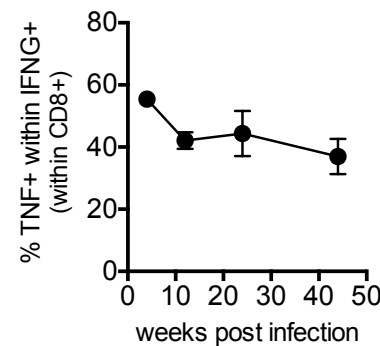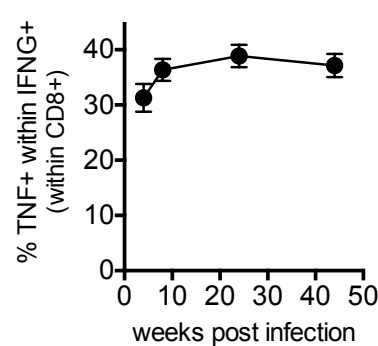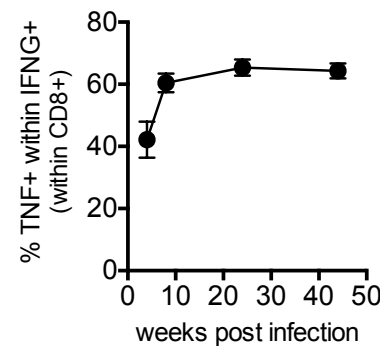

Experiment 'A'

Experiment 'B'

Experiment 'C'

Supplement: S1 Fig — Three similar experiments (‘A’, ‘B’, ‘C’), all which show the kinetics of IFNγ and IL-2 production by pulmonary CD4+ and CD8+ T cells from M. tuberculosis infected mice. Lung mononuclear cells were prepared and stimulated with ESAT6 or TB10 peptide epitopes, or anti-CD3/38 mAbs in vitro. As described in the methods, TNF or IFNγ were detected by intracellular cytokine straining. ‘%TNF+ within IFNγ+’ refers to the ratio of TNF+IFNγ+ cells to total IFNγ+ cells. This ratio provides a functional assessment of the T cells, independently of the number of T cells at anytime point. Each point represents the mean +/- SEM for 3–6 mice/time point. (PDF) [file ppat.1005490.s001.pdf]

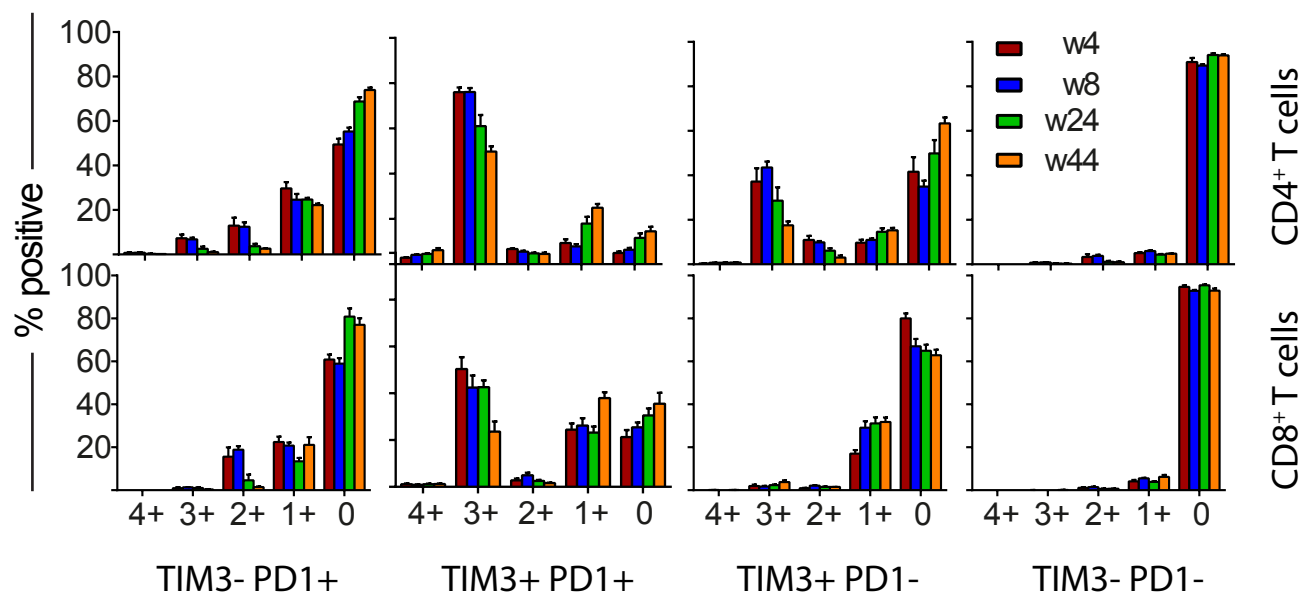

Supplement: S3 Fig — T cells were obtained from lungs of M. tuberculosis infected mice at various time points after infection (2, 8, 24, or 44 weeks) (n = 4–5 per group per time point). CD4+ and CD8+ T cells expressing Tim3 and/or PD1 were analyzed for their expression of other inhibitory receptors (LAG3, CTLA4, CD160, 2B4). 80% of TIM3+PD1+ CD4+ T cells co-expressed three other inhibitory receptors. This frequency was greater than TIM3+PD1– (~40% of cells included 3 other inhibitory receptors) and TIM3—PD1+ CD4+ T cells (<20% of T cells included three other inhibitory receptors). In contrast TIM3–PD1- T cells frequently did not express other inhibitory receptors, regardless of the time point analyzed. Data are representative of 2 independent experiments. (PDF) [file ppat.1005490.s003.pdf]

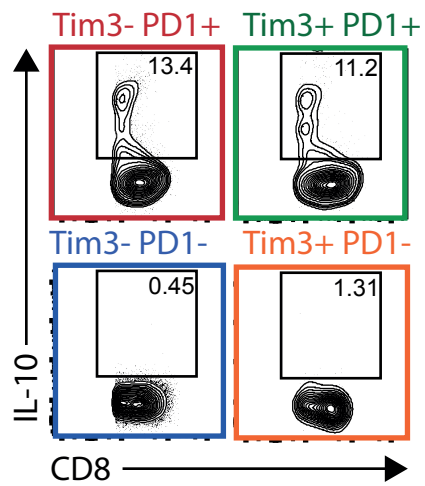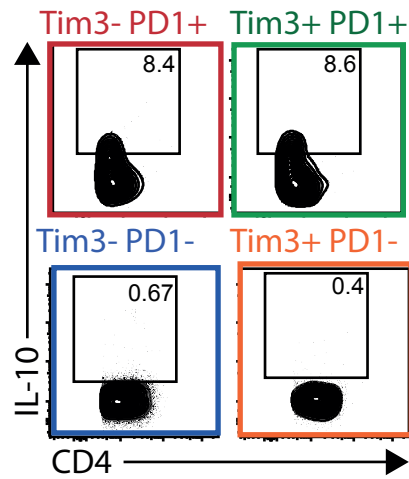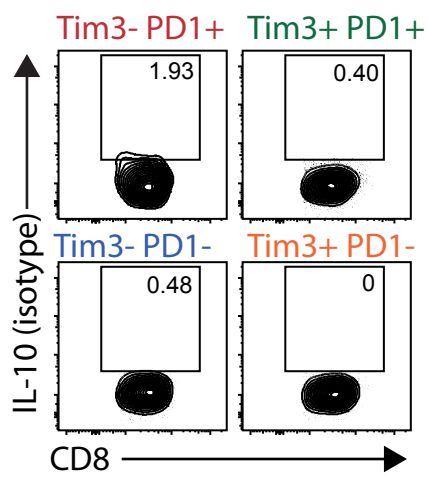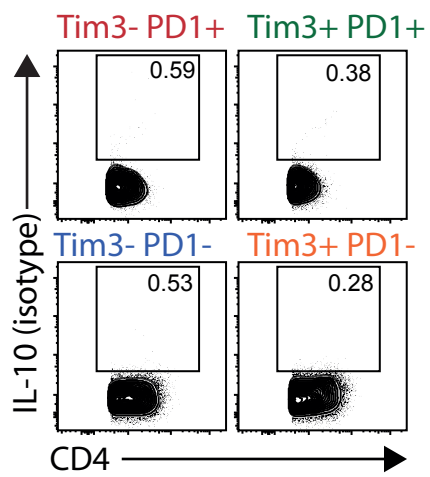

Supplement: S4 Fig — Representative flow cytometry plots of intracellular IL-10 production by TIM3- and PD1-expressing CD4+ (right panels) and CD8+ (left panels). T cells from the lungs of chronically M. tuberculosis infected mice were stimulated in vitro with anti-CD3/28 mAbs. An antibody specific for IL-10 (upper panels) or an isotype control (lower panels) was used for intracellular staining. Data are representative of 2 independent experiments, each with 3–4 mice per group. (PDF) [file ppat.1005490.s004.pdf]

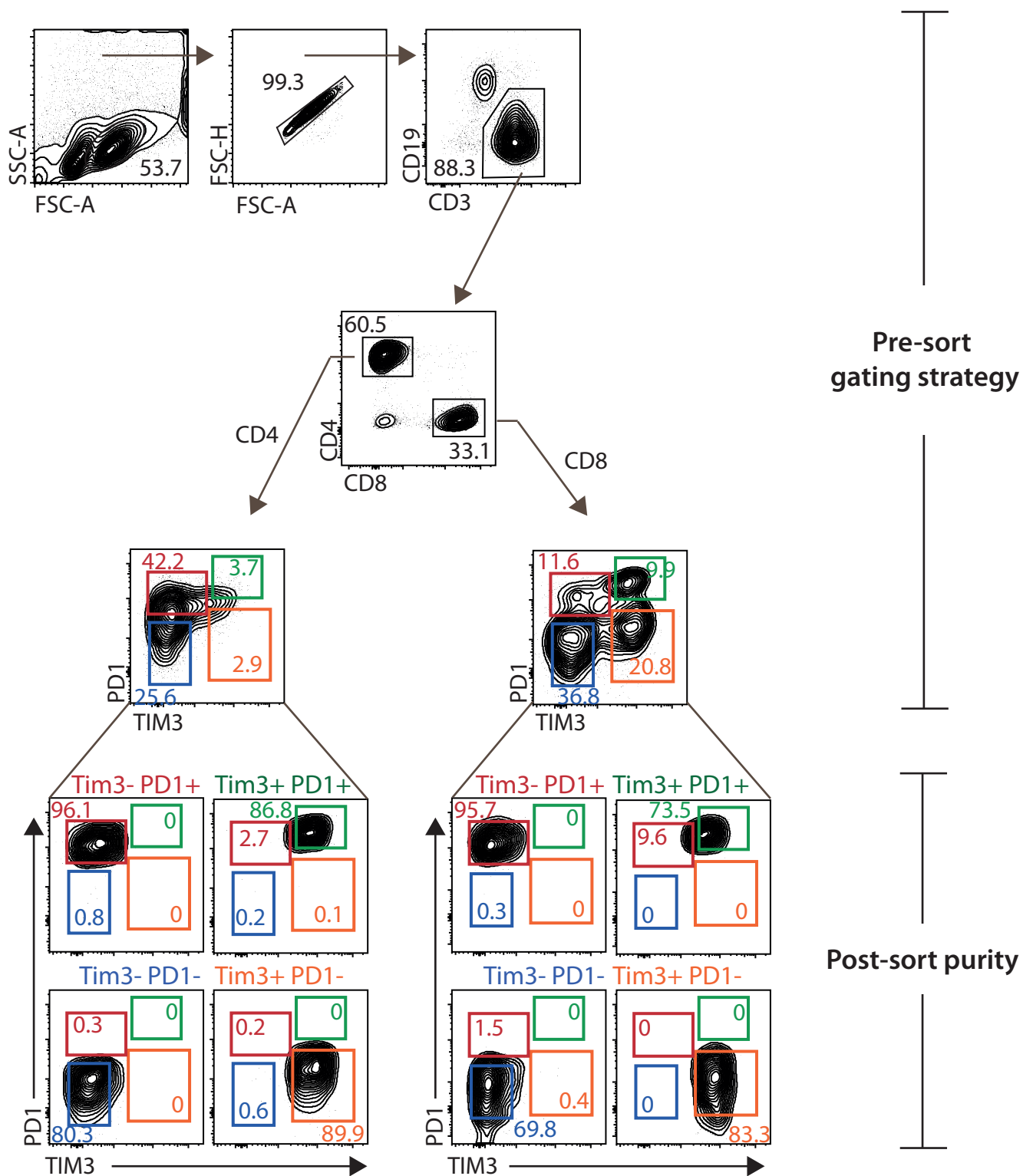

Supplement: S5 Fig — Lung mononuclear cells were obtained by collagenase digest and T cells were enriched by negative selection using immunomagnetic beads. Lymphocytes were identified based on size and scatter, and after gating on singlets, CD4+ or CD8+ T cells were identified based on CD3+CD4+ or CD3+CD8+ expression. For each population of CD4+ or CD8+ T cells, four Tim3- and PD1-expressing populations were sorted: (1) Tim3–PD1+, (2) Tim3+PD1+, (3) Tim3+PD1–, (4) Tim3–PD1–. A sample of each sorted population was reanalyzed to verify the phenotype assess the purity before performing Nanostring analysis. (PDF) [file ppat.1005490.s005.pdf]
