# Supplementary material for: TIM3 Mediates T Cell Exhaustion during Mycobacterium tuberculosis Infection
Source: PLoS Pathog. 2016 Mar 11;12(3):e1005490. doi: 10.1371/journal.ppat.1005490 (PMC4788425; doi:10.1371/journal.ppat.1005490)
Supplement: S2 Fig — (A) Lung mononuclear cells were purified from the lungs of mice 18 days after M. tuberculosis infection and stimulated in vitro with ESAT61-15 or TB10.44−11 peptide, anti-CD3/CD28 mAb, or media (unstimulated) control. Representative FACS plots of CD4+ or CD8+ T cell stained for intracellular IL-2 and IFNγ. (B) Three similar experiments (‘A’, ‘B’, ‘C’), all which show the kinetics of IFNγ and IL-2 production by pulmonary CD4+ and CD8+ T cells from M. tuberculosis infected mice. The frequency of ESAT6-specific CD4+ or TB10.4-specific CD8+ T cells that produce IL-2 or IFNγ after stimulation in vitro with peptide epitopes and intracellular cytokine staining. (C) The fraction of CD4+ and CD8+ T cells producing different combinations IFNγ, TNF or IL-2 after in vitro stimulation with peptide epitopes or anti-CD3/CD28 mAb. Each pie slice represents the fraction of the total CD4+ or CD8+ T cell cytokine response that produces the combination of cytokines indicated in the legend. (PDF) [file ppat.1005490.s002.pdf]

A.

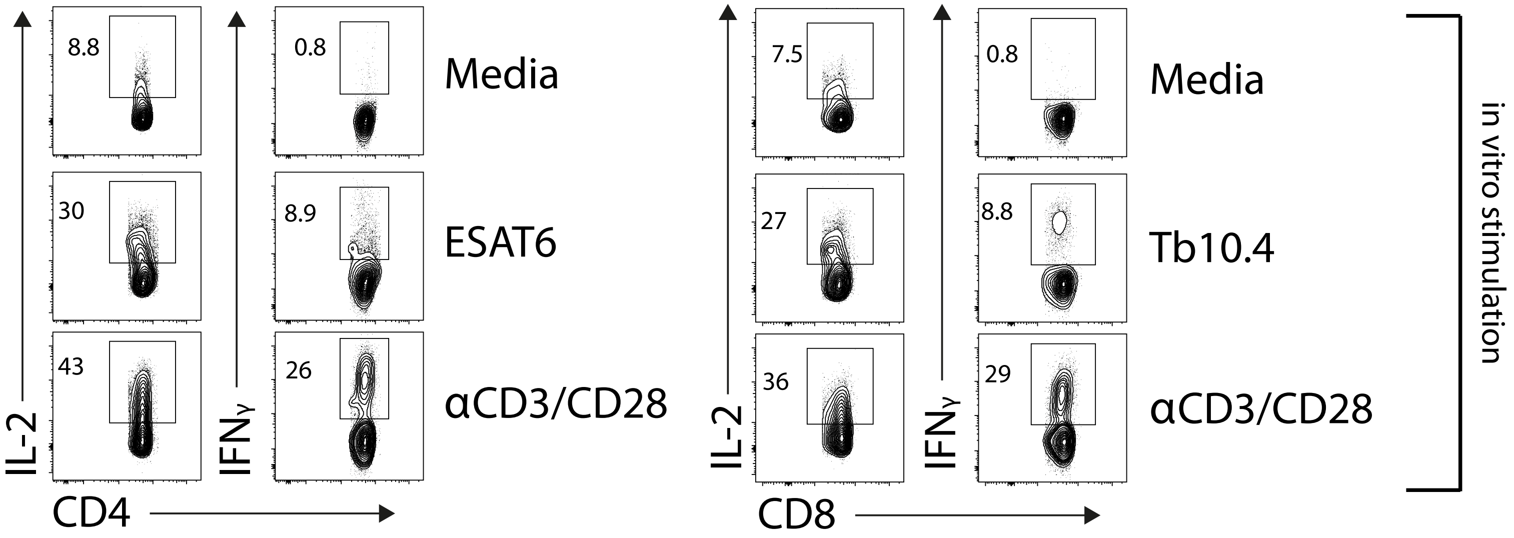

B.

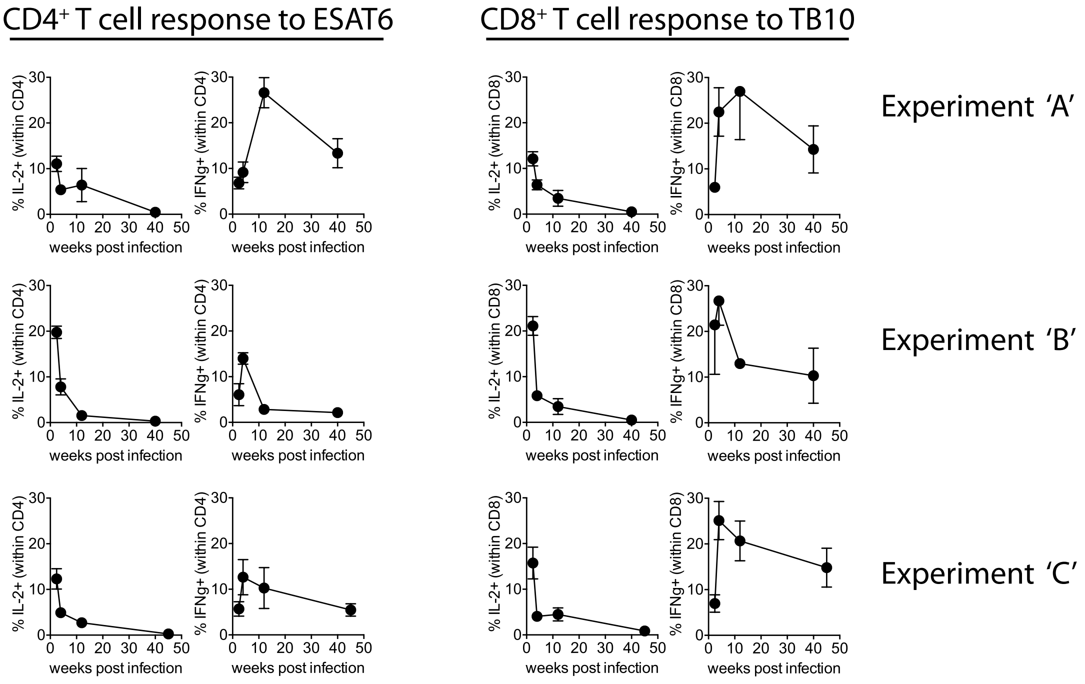

C.

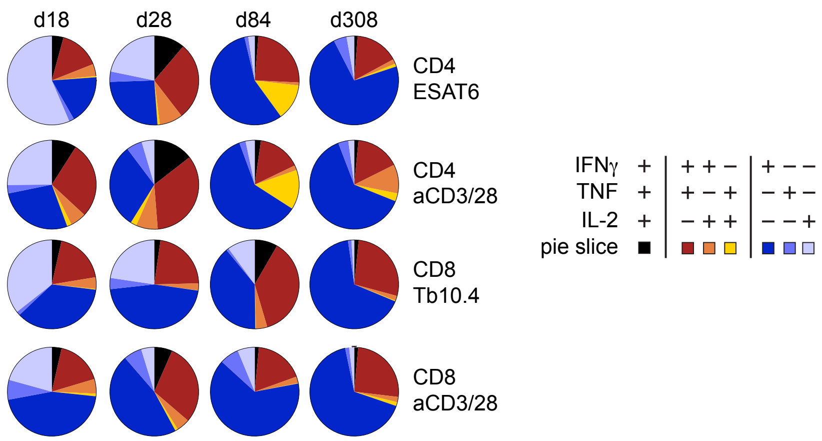

Suppl Fig 1
