# Supplementary material for: TIM3 Mediates T Cell Exhaustion during Mycobacterium tuberculosis Infection
Source: PLoS Pathog. 2016 Mar 11;12(3):e1005490. doi: 10.1371/journal.ppat.1005490 (PMC4788425; doi:10.1371/journal.ppat.1005490)

A. TIM-3 expression by pulmonary myeloid cells from uninfected mice

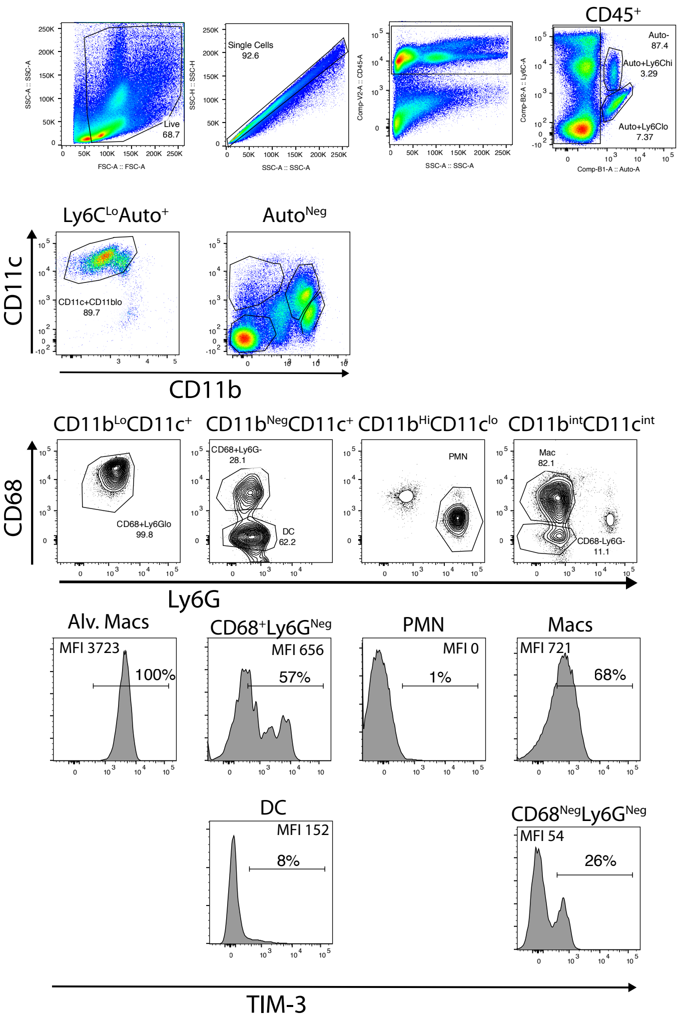

B. TIM-3 expression by pulmonary myeloid cells from Mtb infected mice

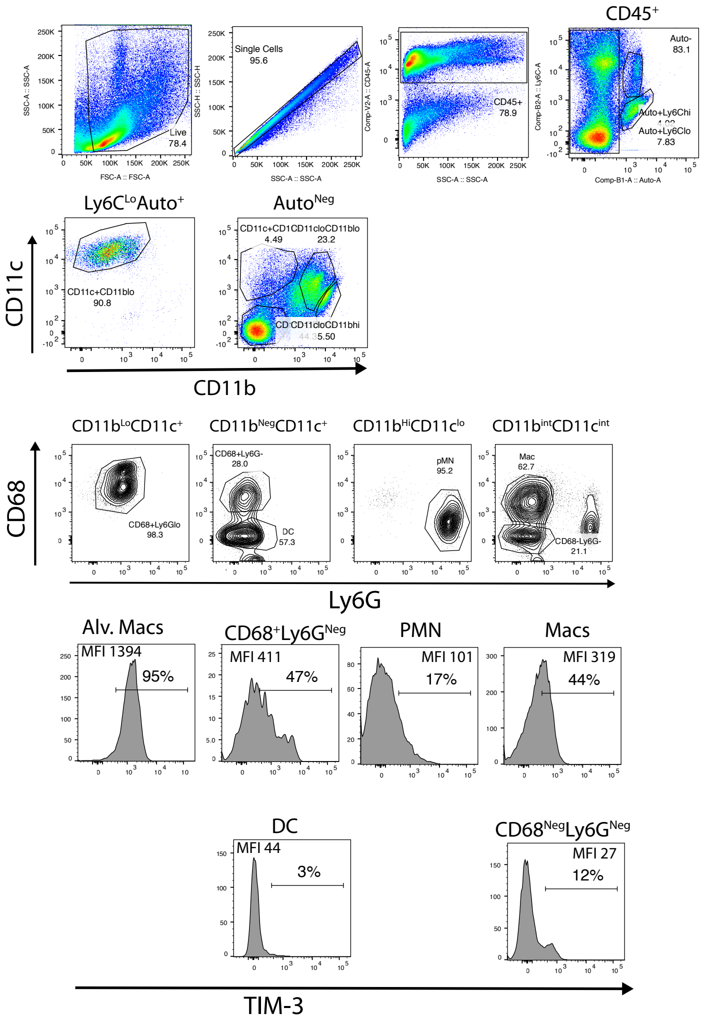

Supplement: S6 Fig — Gating strategy for identifying myeloid population Tim3 expression. Representative flow cytometry plots from lungs of uninfected mice (A) and lungs of M. tuberculosis infected mice 21 days post infection (B). Cells of hematopoietic lineage were identified with CD45, then alveolar macrophages were gated on auto-fluorescence. Dendritic cells, recruited macrophages, and neutrophils were identified by CD11c, CD11b, and Ly6G expression. Having identified these various cell types, TIM3 expression by alveolar macrophages, dendritic cells (DC), and neutrophils was determined. TIM3 expression was quantitated as the percentage of positive cells and median fluorescent intensity (MFI). (PDF) [file ppat.1005490.s006.pdf]
